# Editorial: *Pseudomonas aeruginosa* Pathogenesis: Virulence, Antibiotic Tolerance and Resistance, Stress Responses and Host-Pathogen Interactions

**DOI:** 10.3389/fcimb.2022.860314

**Published:** 2022-02-15

**Authors:** Elio Rossi, Melanie Ghoul, Ruggero La Rosa

**Affiliations:** ^1^ Department of Biosciences, University of Milan, Milan, Italy; ^2^ Department of Zoology, University of Oxford, Oxford, United Kingdom; ^3^ The Novo Nordisk Foundation Center for Biosustainability, Technical University of Denmark, Kgs. Lyngby, Denmark

**Keywords:** *Pseudomonas aeruginosa*, antimicrobial resistance (AMR), host-pathogen interaction, bacterial persistence, virulence

*Pseudomonas aeruginosa* is an environmental Gram negative bacterium able to colonize a wide variety of natural environments (Crone et al., 2020). In addition, it is a major cause of nosocomial infections in immunocompromised patients, often associated with acute bloodstream infections, pneumonia, and sepsis, and it is considered the primary cause of death in patients affected by chronic pathologies such as cystic fibrosis (CF), chronic obstructive pulmonary disease (COPD), HIV and cancer.

Its large genome encodes multiple intrinsic determinants that make this bacterium a formidable opportunistic pathogen. Infection management and treatment is difficult, and is worsened by the rapid spread of antimicrobial resistance among *P. aeruginosa* populations; limiting the efficacy of antibiotics. Therefore, non-conventional treatments based on a renewed and deep understanding of *P. aeruginosa* pathophysiology are critical to keep this pathogen under control.

In this Research Topic, we collected different contributions that cover and advance crucial aspects of *P. aeruginosa* pathogenesis. Each of these contributions are an example of the complexity of *P. aeruginosa* pathophysiology and serve as a basis for devising future strategies to treat its infections.

Intrinsic antibiotic resistance and tolerance, and the genomic plasticity that allows easy acquisition of genetic determinants of antibiotic resistance can be considered the most concerning aspects of *P. aeruginosa* infections. Carbapenems represent a primary therapeutic option for patients infected with multidrug-resistant *P. aeruginosa*. Understanding and monitoring the diffusion of genetic resistance determinants, in particular β-lactamases, is critical and necessary to improve antimicrobial stewardship (Yoon and Jeong, 2021). In their work, Yu et al. characterize complex events of transposition and homologous recombination promoting the assembly and integration of mobile elements carrying resistance genes into clinical *P. aeruginosa* isolates, highlighting the widespread diffusion of β-lactamases among *P. aeruginosa* populations. While for other microorganisms survival in the presence of antibiotics largely depends on acquisition of resistance genes, in *P. aeruginosa*, resistance derives from complex physiological and regulatory layers that Langendonk et al. capture and dissect in their review work. These layers include surface proteins, efflux pumps, lipopolysaccharide modifications, resistance genes encoded in the core genome, and the ability to reshape cell physiology to maintain metabolically costly mutations conferring antibiotic resistance (Langendonk et al.). The review work highlights that the resistance layers leading to *P. aeruginosa* success are the main obstacle preventing the development of new treatments and the implementation of long-term therapies. However, the same layers can be exploited as drug targets. Indeed, antibiotic resistance or intrinsic tolerance mechanism can be modulated using molecules that, for example, regulate efflux pumps or counteract the activity of β-lactamases (Langendonk et al.). Similarly, antibiotic susceptibility can be affected by acting on biofilm formation, quorum-sensing and motility, and new potential drugs targeting these processes are in development and reviewed in the work of Langendonk et al.. These treatments are real alternatives to classic antibiotics, and by affecting fitness determinants rather than cell viability directly, are considered less likely to foster the emergence of genetic resistance, and, at the same time, have the benefit to expand the number of druggable targets. Indeed, non-antibiotic therapies are also effective if the pathogenicity determinants are targeted indirectly. For example, small metabolites and interference with cell metabolism is a promising strategy. The work of Fraser-Pitt et al. shows that cysteamine, an aminothiol produced in the metabolism of co-enzyme A, can inhibit the production of several virulence traits, including HCN and biofilm, partially interfering with the glycine cleavage system (Fraser-Pitt et al.). Similarly, Alford et al. identify a potential therapeutic target in the NtrBC two-component regulator system. Nitrogen source availability and NtrBC activity affect biofilm formation and the sensitivity to ciprofloxacin. Further, *ntrBC* mutants show a decrease in their overall virulence resulting in a reduced colonization of mice airways but not of the animal skin. Interestingly, genetic manipulation of the *ntrBC* regulon increase bacterial cytotoxicity (Alford et al.). These results highlight the potential for the identification of new therapeutic targets in cell metabolism, but also reiterate the observation that universal treatments might be difficult to achieve, as virulence trait expression might be conditional and influenced by the environmental conditions and the site of infection.

Targeting the interactions with the host is another approach to fight *P. aeruginosa*. Li et al. show that the flagellar hook protein FlgE can interact with ectopic ATP synthase of epithelial cells. While it is well known that flagella are one of the most immunogenic structures in bacteria (Mahenthiralingam et al., 1994) and a mediator of cell-to-cell adhesion, authors show that FlgE interaction with the ATP5B protein might represent a new pathogenicity mechanism that directly impacts microvascular permeability and access to blood stream (Li et al.).

Finally, novel therapeutics can also stem from our understanding of *P. aeruginosa* interactions with other microbes. *P. aeruginosa* is known to outcompete the normal flora, maintaining dominance over other microorganisms. *Aspergillus fumigatus* is frequently co-isolated with *P. aeruginosa* from the lungs of cystic fibrosis patients. Sass et al. show that antifungal factors produced by *P. aeruginosa* are conditional. While in liquid cultures, *A. fumigatus* growth is inhibited by the presence of the iron chelators pyoverdine and pyochelin (Sass et al., 2018), on solid medium *P. aeruginosa* inhibition of *A. fumigatus* growth depends on the production of elastase and rhamnolipids (Sass et al.). These results suggest again the critical role of environmental conditions in the expression of pathogenicity determinants (Bjarnsholt et al., 2021), a concept that should be considered in the development of future treatments.

*P. aeruginosa* pathophysiology is rich and complex, new pathogenicity mechanisms as those described here are about to be uncovered and provide a new set of targets that can serve as the basis for developing future therapies.

## Author Contributions

ER wrote the initial draft. All authors reviewed the article and approved the submitted version.

## Funding

RLR acknowledge the fundings from the “Cystic Fibrosis Foundation” (CFF), grant number MOLIN18G0, the “Cystic Fibrosis Trust”, Strategic Research Centre Award—2019—SRC 017, by the “Novo Nordisk Foundation Center for Biosustainability (CfB)” grant number NNF10CC1016517 and by the "Independent Research Fund Denmark/Natural Sciences" grant number 9040-00106A.

## Conflict of Interest

The authors declare that the research was conducted in the absence of any commercial or financial relationships that could be construed as a potential conflict of interest.

## Publisher’s Note

All claims expressed in this article are solely those of the authors and do not necessarily represent those of their affiliated organizations, or those of the publisher, the editors and the reviewers. Any product that may be evaluated in this article, or claim that may be made by its manufacturer, is not guaranteed or endorsed by the publisher.
